# Ewing Sarcoma of the Kidney: A Rare Entity

**DOI:** 10.1155/2014/283902

**Published:** 2014-01-09

**Authors:** Maria Fernanda Arruda Almeida, Madhavi Patnana, Brinda Rao Korivi, Neda Kalhor, Leonardo Marcal

**Affiliations:** ^1^Diagnostic Radiology Resident, A. C. Camargo Hospital, Sao Paulo, SP, Brazil; ^2^Department of Diagnostic Radiology, The University of Texas M. D. Anderson Cancer Center, 1515 Holcombe Boulevard, Houston, TX 77030, USA

## Abstract

Ewing sarcoma and primitive peripheral neuroectodermal tumor (PNET) are high-grade malignant tumors typically found in children and adolescents. These tumors belong to the family of small round cell tumors and are of neuroectodermal origin. Primary Ewing sarcoma of the kidney is rare and because of that is an infrequent differential diagnosis in urologic malignancies. Renal PNET mostly presents with nonspecific symptoms such as hematuria and abdominal pain. The imaging findings are uncharacteristic. The diagnosis is based on the histology, immunohistochemistry, and molecular pathologic findings. Once PNET has been diagnosed, multimodal treatment is indicated. Despite all treatment options, the prognosis of those with metastatic disease is poor.

## 1. Introduction

Ewing sarcoma and primitive peripheral neuroectodermal tumor (PNET) were originally described as two distinct pathologic entities, although both share common stem-cell precursor and unique chromosomal abnormality. Because of their similar histologic and cytogenetic characteristics, these tumors are now considered part of a spectrum of neoplastic diseases now known as Ewing's sarcoma family tumors (ESFT), which also includes other malignancies [1–3]. The ESFT are most common in bone. Extraskeletal ESFT are less common and can affect the skin, soft tissue, or viscera [4, 5].

Renal primary sarcomas are a rare group of renal tumors. Ewing sarcoma/PNET of the kidney is distinctly rare, with more than 100 cases reported globally. Among these, leiomyosarcoma is the most common (40–60%) followed by liposarcoma (10–15%) [2, 3].

## 2. Case Report

**Figure 1:**
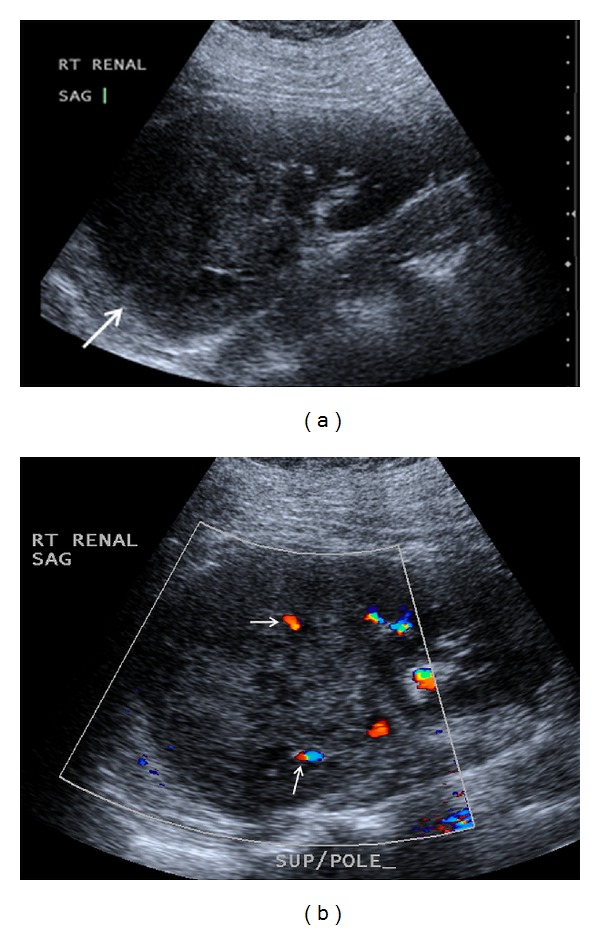
(a) Sagittal ultrasound image of the right kidney shows a large mass replacing the upper pole of the right kidney (arrow). The echotexture is similar to renal parenchyma with mild internal heterogeneity and a few cystic spaces. (b) Sagittal ultrasound image with Doppler shows internal vascularity (arrows) within the large mass replacing the superior pole of the right kidney.

**Figure 2:**
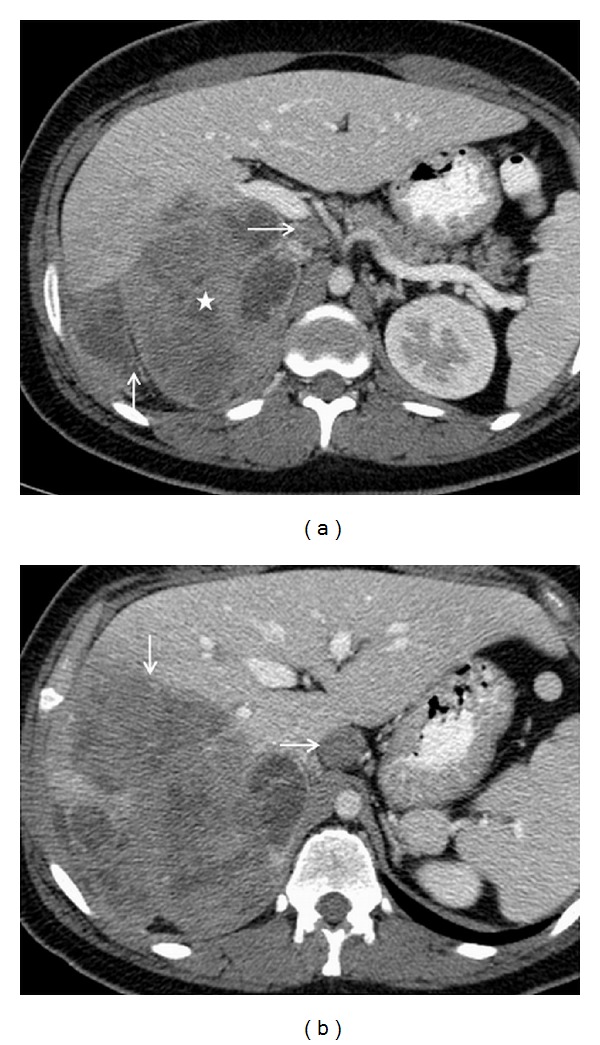
(a) Axial contrast-enhanced multidetector computed tomography (MDCT) image of the upper abdomen reveals a large mass of heterogeneous attenuation replacing the midportion and superior pole of the right kidney (star). Mass has broken into the hepatorenal space (vertical arrow) and invaded the right liver. A metastatic pericaval lymph node is seen (horizontal arrow). (b) Axial contrast-enhanced MDCT image shows a large lobulated component of the renal mass invading the right liver (horizontal arrow). An enlarged metastatic left gastric lymph node is also noted (vertical arrow).

**Figure 3:**
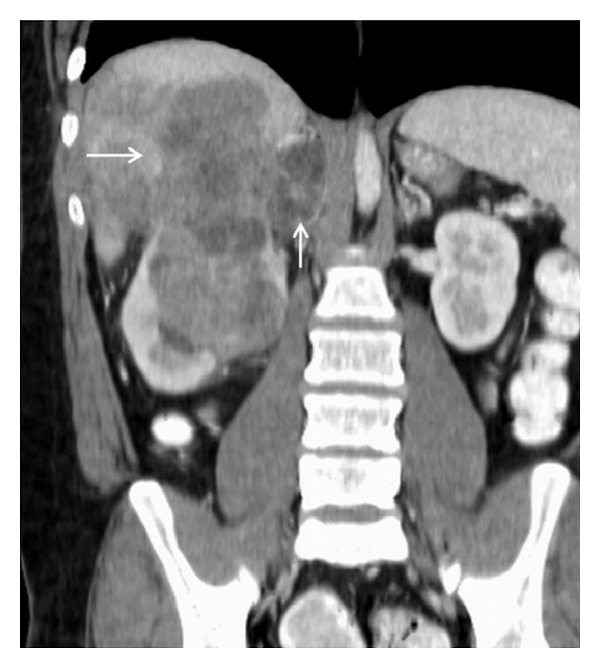
Coronal reconstructed CT image shows large lobulated mass of heterogenous attenuation invading the right liver (horizontal arrow). The right adrenal gland is expanded and completely replaced by the tumor (vertical arrow).

**Figure 4:**
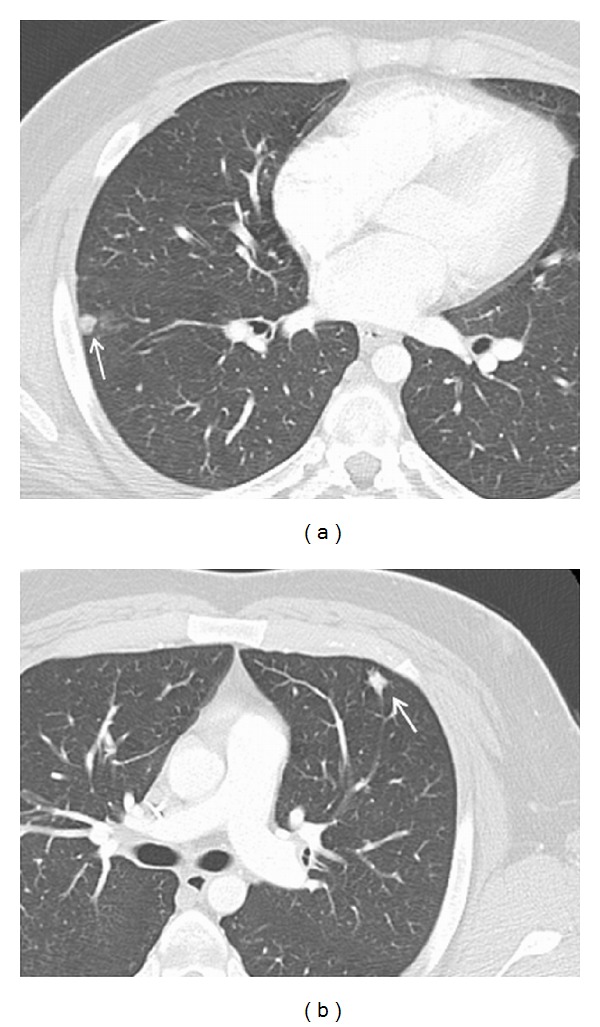
((a) and (b)) Axial CT scan images of the chest reveal small subcentimeter nodules in the right lower lobe (arrow in (a)) and left upper lobe nodule (arrow in (b)), suspicious for pulmonary metastases.

**Figure 5:**
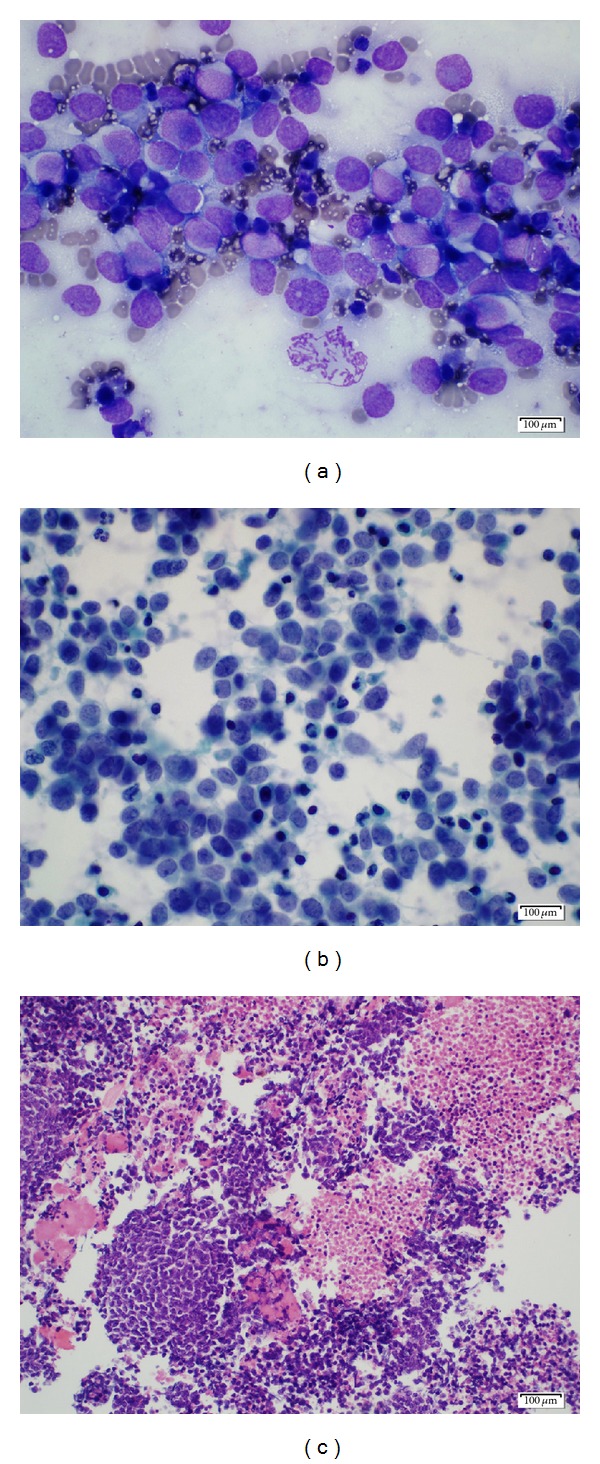
(a) A Diff-Quick stained, air-dried direct smear of Ewing's sarcoma shows a monotonous population of tumor cells with high nuclear: cytoplasmic ratio. Scattered apoptotic cells in the background give an appearance of “double population.” (b) Pap-stained, Alcohol fixed direct smear of Ewing's/PNET shows a malignant small round cell tumor, singly and in loosely cohesive clusters. Numerous apoptotic cells are present. The tumor cells demonstrate mild to moderate nuclear pleomorphism, irregular nuclear contour with fine chromatin, and inconspicuous nucleoli. (c) Pap-stained, alcohol fixed direct smear of Ewing's/PNET shows a malignant small round cell tumor, singly and in loosly cohesive clusters. Numerous apoptotic cells are present. The tumor cells demonstrate mild to moderate nuclear pleomorphism, irregular nuclear contour with fine chromatin, and inconspicuous nucleoli.

A 19-year-old man presented with sudden sharp right flank pain, accompanied by low-grade fevers and vomiting. He had no other significant medical history and denied any episodes of gross hematuria. Due to worsening of the symptoms, an abdominal ultrasound was performed, which showed an infiltrative mass in the upper pole of the right kidney, invading the right liver, concerning for malignancy (Figure 1). A staging CT scan of the abdomen was then performed revealing a right upper pole renal infiltrative mass, invading the inferior aspect of the right lobe of the liver (Figures 2 and 3). The CT scan of the chest revealed a 7 mm left upper lobe nodule as well as tiny punctate nodules in the right lower lobe, suspicious for metastatic disease (Figures 4(a) and 4(b)). Biopsy of the kidney mass revealed Ewing sarcoma/primitive neuroectodermal tumor (Figure 5). Diagnosis was confirmed by fluorescence in situ hybridization (FISH) technique, which showed translocations involving the EWS locus (EWSR1 gene rearrangement). Patient then initiated treatment with multiagent chemotherapy.

## 3. Discussion

Renal cell carcinoma (RCC) is the most common malignant neoplasm of the kidney that constitutes more than 90% of kidney tumors [2, 4]. Renal sarcomas are rare malignancies that constitute less than 1% of all malignant renal tumors. PNET of the kidney is a very aggressive neoplasm that predominantly affects young adults (mean age of presentation between 28 and 34 years), with a slight male predominance [1–3, 6].

Sarcomas of the kidney usually remain asymptomatic until they are large enough to produce symptoms; the average size at the time of diagnosis varies from 5.5 to 23 cm [2, 6]. The clinical findings are uncharacteristic and patients usually complain about pain (85%), palpable masses (60%), and hematuria (37%) [3, 6, 7].

No specific signs of PNET have been described in ultrasonography, computed tomography (CT), or magnetic resonance imaging (MRI). The imaging characteristics of most renal sarcomas are indistinguishable from those of RCC [1]. These tumors appeared as ill-defined, large heterogeneous masses with necrotic and hemorrhagic areas. Using ultrasonography, the tumor appears isoechogenic or hyperechogenic to the renal parenchyma. The CT findings include areas of internal hemorrhage or necrosis, peripheral hypervascularity, and diffuse calcification. On MRI, the tumor appeared isointense or hypointense on T1-weighted images but heterogeneous intermediate to high T2 signal intensity [2, 3]. Venous extension into the renal vein, inferior vena cava, and right heart has been described [3, 5, 8].

The differential diagnoses include Wilms tumor, neuroblastoma, renal cell carcinoma, malignant lymphoma, metastatic renal involvement from sarcoma elsewhere in the body, and renal involvement by a primary retroperitoneal sarcoma [2, 3].

The diagnosis of PNET is based on the pathologic findings, assisted by immunocytochemistry and/or molecular analysis. The pathologic findings show multiple areas of necrosis and hemorrhage. In some cases, the tumor exhibits calcifications and can be surrounded by a fibrous capsule. Overexpression of the surface membrane protein CD99 is a universal feature of EFTs but not pathognomonic because it is also found in synovial sarcomas and gastrointestinal stromal tumors [3, 9].

In the case of a large renal tumor with necrotic and hemorrhagic areas, fine needle aspiration is a valuable diagnostic tool [3]. Image-guided renal biopsy may play an important role in determining preoperative diagnosis of Ewing sarcoma, so that an appropriate treatment plan which includes preoperative chemotherapy can be developed prior to surgical excision [1].

Ewing sarcoma and PNET exhibit aggressive course characterized by early metastatic disease (25–50% at the time of presentation) and is considered to be a systemic disease. Patients undergoing only local therapy have a recurrence rate of 80% to 90%, presumably because of subclinical metastasis at the time of initial diagnosis [1]. In addition to local lymph node involvement, renal PNET often metastasizes to the lung, liver, and bone [2, 3]. MRI and CT provide an accurate assessment of local resectability and the detection of distant metastases. Furthermore, the use of 99-technetium scintigraphy is a sensitive method for the detection of bone metastases [3].

Successful treatment requires multimodality approach that consists of surgery, chemotherapy, and radiotherapy [3]. Postoperative radiotherapy must be added in the case of inadequate surgical margins. PNETs demonstrate high response (94% complete response) to multimodal approaches. Patients with localized tumor have a 5-year disease free survival rate of approximately 45–55% [4, 6]. Despite aggressive treatment, the prognosis of patients with metastatic disease is poor, with overall cure rate of 20% [3, 6, 10–12]. One of the main challenges is proper diagnosis and adequate treatment in expedited time [13].

## 4. Conclusion

PNET is an infrequent differential diagnosis in urologic malignancies. Renal PNET occurs predominantly in young adults and has a tendency to be extremely aggressive. Patients often have nonspecific symptoms such as hematuria and abdominal pain. The CT and MRI findings are nonspecific, but they are useful methods for local assessment of resectability and detection of metastases. The diagnosis is based on the histologic, immunohistochemistry, and molecular pathologic findings. Image-guided renal biopsy may play an important role in determining preoperative diagnosis. Once PNET has been diagnosed, multimodal treatment is indicated. Despite all treatment options, the prognosis is poor.
